# Supply chain resilience and industry 4.0: a evaluation of the Brazilian northeast automotive OEM scenario post COVID-19

**DOI:** 10.1186/s42467-021-00010-1

**Published:** 2021-08-02

**Authors:** Milton C. Soares, Cristiano V. Ferreira, Thiago B. Murari

**Affiliations:** 1 PPG GETEC, University Centre SENAI CIMATEC, Av. Orlando Gomes, 1845, Salvador, 41650-010 Brazil; 2 Technological Center of Joinville, Federal University of Santa Catarina, Rua Dona Francisca, 8300, Joinville, 89219-600 Brazil

**Keywords:** Industry 4.0, Outbreak, Resilience

## Abstract

COVID-19 outbreak has heavily impacted the manufacturing industry, including Brazilian Automotive Industry. The effects of COVID-19 created restrictions in several industry processes as supply chain. On the other hand, several industry 4.0 technologies is able to support the industry supply chain activities in the COVID 19 scenarios, as well it may contributed for the automotive industry recovery and it will define the next steps of this industry. A supply chain is a network between a company and its suppliers to produce and distribute a specific product to the final buyer. Industry 4.0 is related to the technology development and the digitalization process that improve significantly productivity. Considering the automotive process, an important reference model is described in Advanced Product Quality Planning and Control Plan, that is a manual that communicate the guidelines of the product quality planning and control plan for internal and external suppliers. In this scenario, this paper evaluated the current situation and the future outlook for the adoption of Industry 4.0 technologies in the automotive OEM post-pandemic scenario on the point of view of automotive specialists. The results of this research provide an overview of the current situation and the future outlook for the usage of Industry 4.0 technologies by the Brazilian Northeast automotive OEM, from the perspective of manufacturing engineering experts on APQP.

## Introduction

Coronavirus disease 2019 (COVID-19) is an outbreak of respiratory illness caused by Sars-CoV-2 virus and firstly reported in China [1]. It was declared pandemic by the World Health Organization on March 11, 2020 [2]. Thenceforth COVID-19 outbreak has heavily impacted the manufacturing industry. Original Equipment Manufacturer (OEM) and parts suppliers have yet to return to full production capacity. For instance, Aston Martin Lagonda Global Holdings Plc plan to eliminate its workforce by 20% [3] and General Motors have slowed the manufacturing plants because of safety and lockdown protocols [4]. Brazilian automakers cancelled their quarantine and returned to manufacture vehicles, even with an ongoing outbreak, according to the Brazilian Association of Automotive Vehicle Manufacturers (ANFAVEA) guide protocols [5]. But considering COVID-19 contingency mode that creates restrictions to the manufacturing floor, manufacturing processes, review meetings and visits to suppliers to keep the social distance in order to avoid contamination, this may change the way that the OEM’s deals with the suppliers.

The automotive industry has a huge contribution in the economy of the country, contributing positively to the generation of jobs and whose financial results are used to measure the current wealth of the international economy [6]. For instance, it represents about 5% of the Brazilian gross domestic product (GDP) and approximately 20% of the GDP of the manufacturing industry [7], with 26 manufactures, 484 autoparts and 5279 car dealers employing more than 1.3 million workers around the country [8]. But the OEMs and their supply chain are currently working in a contingency mode. A slump in car sales were projected in 2020, predicting 64 million automobiles to be sold worldwide, against 80 million estimated pre-pandemic [9]. This scenario demands Supply Chain Resilience (SCR), defined as the ability to prevent and absorb changes, recovering initial performance after an unexpected disruption [10]. Following a disruptive event, key players in supply chain must predict, understand and to be prepared for the impact in this event. They need to define strategies to respond quickly and to adapt to the resulting effects, as well as rearrange your resources to strengthen skills [11].

COVID-19 is an unprecedented disruptive event in recent decades. In the current state, the lack of important information is creating a huge obstacle to respond to the interruption caused by this pandemic, which leads to a reactive and disorganized response to these interruptions, further compromising the SCR [12]. This outbreak clearly shows the need for improve the SCR research and practices [13].

The aim of this paper is to present a method to evaluate the current situation and the future outlook for the adoption of Industry 4.0 technologies in the automotive OEM post-pandemic scenario. A survey was applied to capture the perception of automotive manufacturing specialists regarding how the Industry 4.0 technologies supports the Advanced Product Quality Planning and Control Plan (APQP) phases. This proposed method may help the automotive supply chain to efficiently implement Industry 4.0 technologies to improve their SCR.

## Industry 4.0

The term Industry 4.0 is related to the technology development and the digitalization process that brought significant productivity improvements. The term was first time introduced by the German government in Hannover Messe of 2011 and later it was declared as a strategic initiative to transform the manufacturing industry [14].

To support the flexibility of demand and personalized products in small batches that has been increasing considerably in the latest times, a combination of several digital technologies like; Artificial Intelligence, Cloud Computing, Autonomous Robots, Augmented Reality, Additive Manufacturing and Internet of Things (IoT) had to be introduced to allow the connectivity between suppliers, OEMs and costumers. All of this leads to efficiency and productivity improvements that are changing key business processes and increasing the competitive power of organizations [15].

A conceptual framework for Industry 4.0 was proposed based on three core and nine fundamental technologies that are transforming the industrial manufacturing: adaptive robotics, cyber physical infrastructure, sensors and actuators, additive manufacturing, cloud technologies, virtualization technologies (Virtual Reality (VR) and Augmented Reality (AR)), simulation, data analytics and Artificial Intelligence (AI), Real-time Locating Systems (RTLS) and Radio Frequency Identification (RFID) technologies, communication and networking, mobile technologies and cybersecurity [15].

Smart factories are the key feature for the Industry 4.0 and the main sub-processes to support it are listed below [16]:

- Machine-to-Machine communication via IoT
- Consistent communication from the sensor to the cloud
- Integration of robotics and innovative drive technologies
- RFID as the basis for parts tracking and intelligent products

These technologies support the three major advantages of Industry 4.0: vertical integration, horizontal integration and end-to-end engineering [17, 18]. While the vertical integration is related to the integration of information and communications technology systems in different hierarchical levels of an organization [18], the horizontal integration is the collaboration of resources and real time information exchange between enterprises, like supply chain, manufacturing and customer [19]. End-to-end engineering is the integration of engineering from product development to post-sales [18]. The vertical and horizontal integration shows the importance of Supply Chain to implement the Industry 4.0 framework technologies to collaborate and exchange information with the automotive OEM in real time.

### Review of COVID-19 challenges that impact supply chain and industry 4.0 technologies to overcome them

The COVID-19 outbreak is the most serious disruption for the entire supply chain in recent history [12, 13]. Companies still seem to be unprepared in terms of supply chain mapping and visibility to deal with COVID-19 [12, 20]. According to [21], worldwide supply chains connecting the world to China and other manufacturing centers are expected to be seriously disrupted.

[22] identified the operational challenges confronted by retailers in providing efficient services and discussed some Industry 4.0 technologies to mitigate them. According to [22], theirs proposed framework may assist policymakers to develop an action plan for COVID-19. In our understanding, companies can take advantage of the roadmap proposed in this study for plan Industry 4.0 implementation also. Table 1 intend to summarize their findings. We added some other inputs from the available literature regarding the technologies or strategies that may mitigate supply chain issues caused by COVID-19 [13, 23–25].

**Table 1:** Summary of Industry 4.0 technologies that may mitigate supply chain issues caused by COVID-19 [13, 22–25]

Supply Chain Challenges on COVID-19	Industry 4.0 technologies or strategies to mitigate outbreak issues
Lack of supply chain flexibility	Big data analytics [22]
Lack of government support	Blockchain [22]
Lack of trust	Blockchain [22] and Digital connectivity [25]
Communication issues	IoT, Cloud Computing [22] and supply chain automation [25]
Lack of security and safety	Robotic and automated devices [22]
Shortage of manpower	Cloud-based systems (for training and education) [22]
Consumer Behavior	Big data analytics and AI [22]
Lack of balance in supply and demand	Data Analytics [22]
Poor infrastructure	Flexible layout [22]
Lack of medical facilities	AI, blockchain and IoT [22]
Lack of viability	Simulation [13] (i.e. digital supply chain twins [23])
Lack of access	Cybersecurity (high levels of remote access to core systems) [24] and localization of sourcing [25]

In another study, [25] states that the best strategies to mitigate the risks attributed to COVID-19 in automobile industry is to develop localized sources of supply and to use advanced technologies of Industry 4.0, as Big Data Analytics that plays a significant role in providing real-time information on supply chain activities to overcome the challenges created by the pandemic. Moreover, [26] evaluated the impact of COVID-19 outbreak on employee performance of the service sector. They claim that the moderating role of Industry 4.0 technologies is more comprehensive in employee quality performance than delivery performance.

The COVID-19 pandemic is a recent event that needs further studies that seek to understand the main consequences that it has caused in the supply chain of each industrial sector. For instance, the automotive supply chain has more than 900 Tier 1 [27] and several hundred thousand Tier 2 and Tier 3 suppliers [28]. Although the literature reviewed here is not about in-depth studies on the challenges that COVID-19 brought to the supply chain in the automotive industry, we understand that the challenges presented in other sectors of the industry may be used as a basis to assess the future scenario of adoption of Industry 4.0 technologies in Tiers 1 and 2.

## Advanced product quality plan phases

The APQP is a reference manual released by Ford, Chrysler and General Motors in July of 1994. The propose of this manual is to communicate the guidelines of the product quality planning and control plan for internal and external suppliers. Although designed for use in the automotive industry, as required in the QS-9000 manual, APQP is virtually suitable for any product quality planning [29].

The manual splits APQP in 5 phases [29, 30]:

- Phase 1, Plan and Define Program. The decisions to be considered during the first step of the product development must be focused on the consumers expectations and needs based on quality and manufacturing standards. The goal of the first phase of the APQP is to assure that the product program is meeting customer needs while providing competitive value. The goal of any product program is meeting customer needs while providing competitive value. The initial step of the product quality planning process is to ensure that customer needs and expectations are clearly understood. In this phase, resource planning, process and product assumptions are made. A list of preliminary special characteristics and design / reliability goals are also established. Tools which typically provide great benefit in this section are Marketing Research, Historical Warranty and Quality Information, Team Experience, Business Plan, Product Product/Process Benchmark Data and Assumptions, Reliability Studies, Customer Inputs, Design Goals, Bill of Material, Flow Chart, Product Assurance Plan and others.
- Phase 2, Product Design and Development. This phase focus on developing the design of the product. The product must be feasible and meet user expectations also. Some of the outputs in this phase are Design Failure Mode and Effects Analysis, Design Verification, Prototype Build and Control Plan, Engineering and Material Specifications. To explain this phase more clearly, we have split it into three subphases based on the Product Development Process (PDP) [31].
  - Phase 2.0, Informational Project. The quality and manufacturing requirements are considering assuring the feasibility of the manufacturing process attending the customers, government and engineering requirements according to its volumes. These information supports the economic and technical feasibility decisions. The goal of the informational phase is to develop a set of data based on the information raised during the planning and other sources, to drive the criteria to the decisions in the following phases of the product development.
  - Phase 2.1, Conceptual Project. During the conceptual phase the activities of the team are related to the research, creation, representation and define the solutions to the problems of the projects. The research for the solutions already in place can be done through benchmark studies. The creation of the solutions is free from restriction, since the specifications of the projects are being considered. The representation of the solutions cane be done through sketches and manual drawings, in parallel with the creation. The selected solutions are made based on properly methods supported by the requirements defined previously.
  - Phase 2.2, Detailed Project. The goal of this phase is to develop and deliver all project specifications based in the prior work. The project is detailed into engineering drawings and specifications documents.
- Phase 3, Process Design and Development. The manufacturing process development phase defines how the product will be manufactured and assembled based on the technological point of view, this is the time to formalize the process and the additional activities related to the plan and control of the production. The tasks to be accomplished at this phase of the product quality planning process depend upon the successful completion of the prior stages contained in the first two phase. This next step is designed to ensure the comprehensive development of an effective manufacturing system. The manufacturing system must assure that customer requirements, needs and expectations are met. This phase explores manufacturing techniques and measurement methods that will be used to bring the design engineer’s vision into reality. Process Flow Charts, Process Failure Mode and Effects Analysis and Control Plan Methodology, Product/Process Quality System Review, Process Flow Chart, Floor Plan Layout, are examples of tools used in this section.
- Phase 4, Product and process validation. Used to validate the manufacturing process through the production trial run and all the activities related to it. The main goal is to verify if the long-term production process is capable of meeting all the engineering requirements and specifications. Some examples of tools used in this phase are: Statistical Process Control, Measurement Systems Analysis, Process Capability Studies, Product Validation Testing, Packing Evaluation. In this phase, Product Part Approval Process (PPAP) is ready for submission and production begins upon approval. To explain it clearly, we have split it into two subphases based on the Product Development Process (PDP) [31].
  - Phase 4.0, Process validation. In order to validate the product and the process, a trial run is established according the product volumes. During the production of this batch, the manufacturing process and the product are evaluated according the quality and engineering requirements.
  - Phase 4.1, Product Launch. The goal of this phase is to launch the product in the market with the same results of the previous phase to ensure the acceptance of the product by the potential consumers.
- Phase 5, Feedback, assessment, and corrective action. The focus is to guarantee the production of the product in mass production and statistics studies are established based on the customer and engineering requirements. When issues are raised, due either special or common-cause variation, containment and corrective actions are required. The manufacturing and development teams works together to solve the issues on time in order to protect the customers.

Table 2 shows a summary of the relationship between APQP and PDP, as well as the desired outputs in each APQP phase. Considering the phases and tools described in APQP and PDP, there is an perspective to integrate it to the Industry 4.0 technologies. This will be discussed in the following sections.

**Table 2:** Relationship between APQP and PDP for each phase, with outputs

	Phase 1	Phase 2	Phase 3	Phase 4.0	Phase 5
	Plan and Define Program	Product Design and Development	Process Design and Development	Process validation	Feedback, assessment, and corrective action
APQP Phase main goal [30]	Define customer requirements and expectations regarding the product	Deliver the product design, as well as the feasibility assessment	Design and develop the production process considering product specifications, product quality, and production costs	Validate the manufacturing process and the final product	Evaluate and improve processes
PDP macro phases relationship with APQP [31]	Project planning	Informational, conceptual and detailed project	Product manufacturing preparation	Product validation and launch	Monitoring of product and process
Expected Outputs [30]	1. Design goals;	1. Design FMEA;	1. Packaging standards and specifications;	1. Significant production run;	1. Reduced variation;
	2. Reliability and quality goals;	2. DFM/A;	2. Quality system review;	2. MSA results;	2. Improved customer satisfaction;
	3. Preliminary bill of material;	3. Design verification;	3. Process flowchart;	3. Process capability studies;	3. Improved delivery performance;
	4. Preliminary process flow;	4. Design review;	4.Floor plan layout;	4. Production part approval process;	4. Effective use of lessons learned.
	5. Preliminary list of special characteristics;	5. Prototype control plan;	5. Characteristics matrix;	5. Production validation testing;	
	6. Product assurance plan;	6. Engineering drawings CAD the master;	6. Process FMEA;	6. Packaging evaluation;	
	7. Gateway approval.	7. Engineering specifications;	7. Pre-launch control plan;	7. Production control plan;	
		8. Material specifications;	8. MSA plan;	8. Quality planning sign-off and gateway approval.	
		9. Change control for drawings;	9. Preliminary process capability plan;		
		10. New equipment, tooling, and facilities requirements;	10. Gateway approval.		
		11. Special product and process characteristics;			
		12. Gauges/testing equipment requirements;			
		13. Team feasibility commitment and gateway approval.			

## Method

A survey has been developed to show the perception of Industry 4.0 technologies current usage during the APQP phases and also the expert perspective in a scenario of 5 years up from now. The survey had questions regarding the usage level for nine technologies of Industry 4.0 framework [15] on the five phases of the APQP. The nine technologies of Industry 4.0 framework used in this study are listed bellow:

- Adaptive Robotics
- Cyber-Physical Systems
- Additive Manufacturing
- Cloud Technologies
- VR and AR
- AI and Data Analytics
- Communication and Networking - IoT
- RTLS and RFID
- Cybersecurity

Sixteen manufacturing engineers in automotive industry, experts in manufacturing process, answered the survey between December of 2020 and January of 2021. 87.5% of this experts works in 2 automotive OEM from Brazilian Northeast and the same percentage reported that they have more than 10 years of experience in the automotive industry. All of them defined their knowledge in APQP between moderate to high. The knowledge in Industry 4.0 technologies were reported as moderate to high by 81.25% of the participants.

**Fig. 1:**
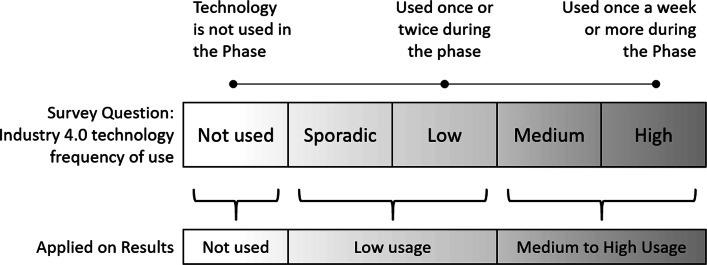
Industry 4.0 technology frequency of use scale. Each survey question presented 5 options for the specialist to choose, ranging from “Not Used” to “High Usage”. Each answer was applied in the evaluation based on the line “Applied on Results”

The survey presented five response categories for each question to be chosen by the specialist according to the current frequency of use of each Industry 4.0 technology from the perspective of manufacturing engineers in a new program. Moreover, the answers were applied into the results based in 3 categories, as shown in Fig. 1.

## Results and discussion

We compiled the answers in graphs to evaluate the empirical results of this survey. We recall that these all results represent the current perceptions and future scenario expectations of some Brazilian Northeast automotive OEM manufacturing specialists for the use of Industry 4.0 technologies on APQP.

**Fig. 2:**
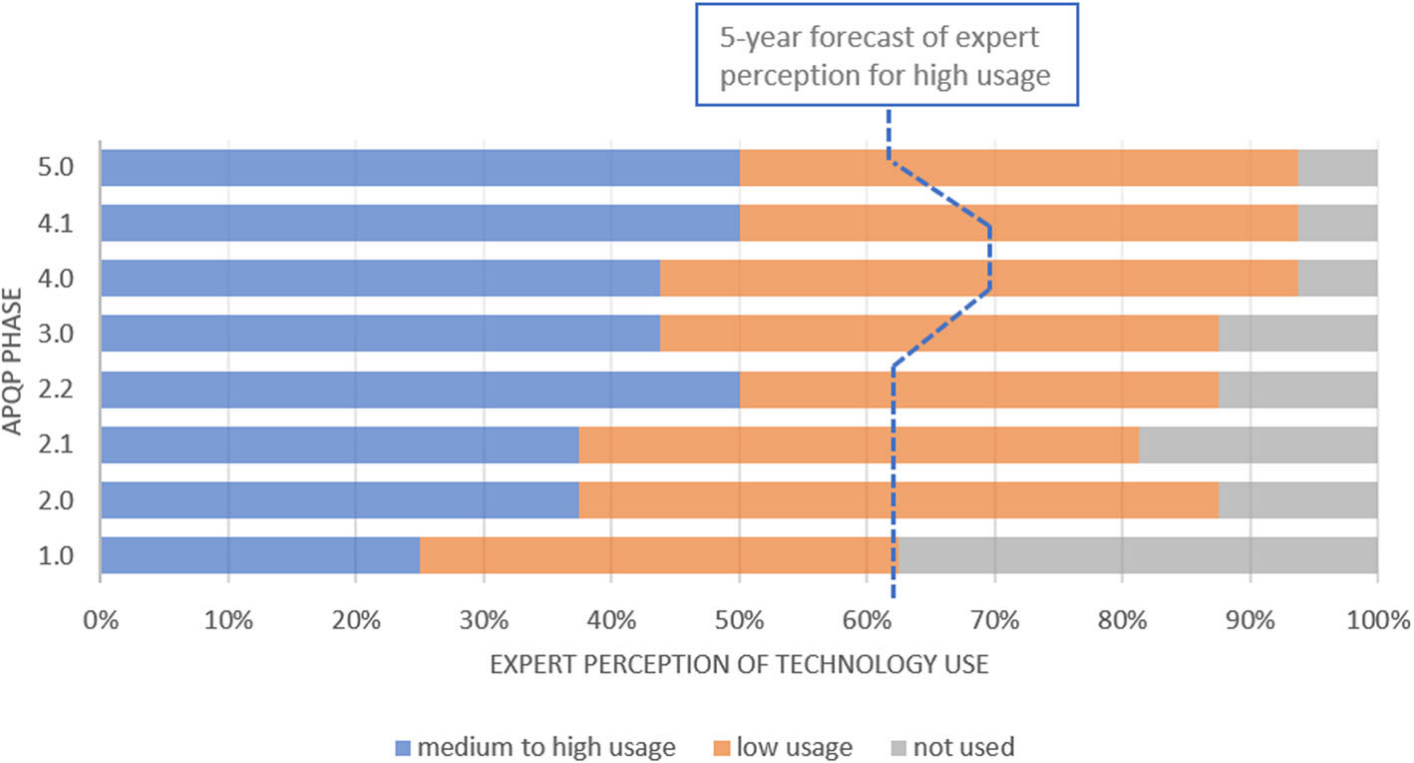
Communication and Networking - IoT. The current technology perception of medium to high use is less expressive at APQP phase 1 and this perception is higher for the next phases, reaching 50% in phases 4 and 5. The forecast shows the amount of experts that believe in the rise of technology use for all phases

In terms of IoT (Fig. 2), the data gathered shows that the specialists believe that it is more utilized during the phases 2.2, 4.1 and 5, what can be understood through the necessity of getting the support of the connectivity between computers and machines to the preparation of the production of the product almost in its final design. For the perspective of 05 years ahead, the IoT will be highly used from the perspective of more than 60% of the interviewed, supporting the validation of the product and process throughout the whole APQP phases.

**Fig. 3:**
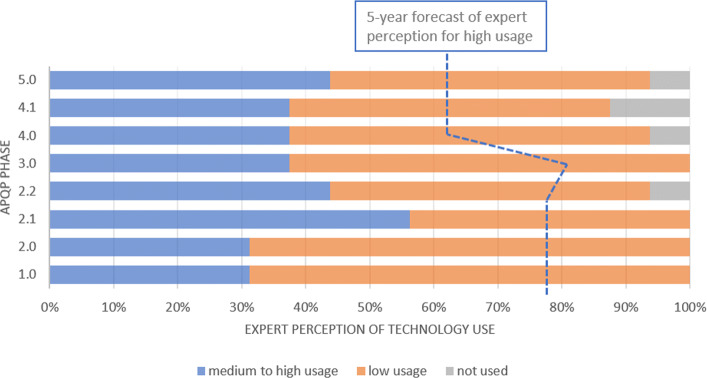
Cloud Technologies This technology is widely used today. In addition, it presents a significant spike in the expected use for the next 5 years

Cloud Technologies are more required on the conceptual phase of the product development (phase 2.1). It is the phase that engineers and designers create sketches to develop engineering solutions for user requirements. More than 60% answered that this technology will be important to all phases of APQP over the next 5 years, highlighting high probability of use in phases 1, 2 and 3 (Fig. 3). Based on the survey, IoT and Cloud are the technologies of Industry 4.0 that are most used today in the automotive OEM industry.

**Fig. 4:**
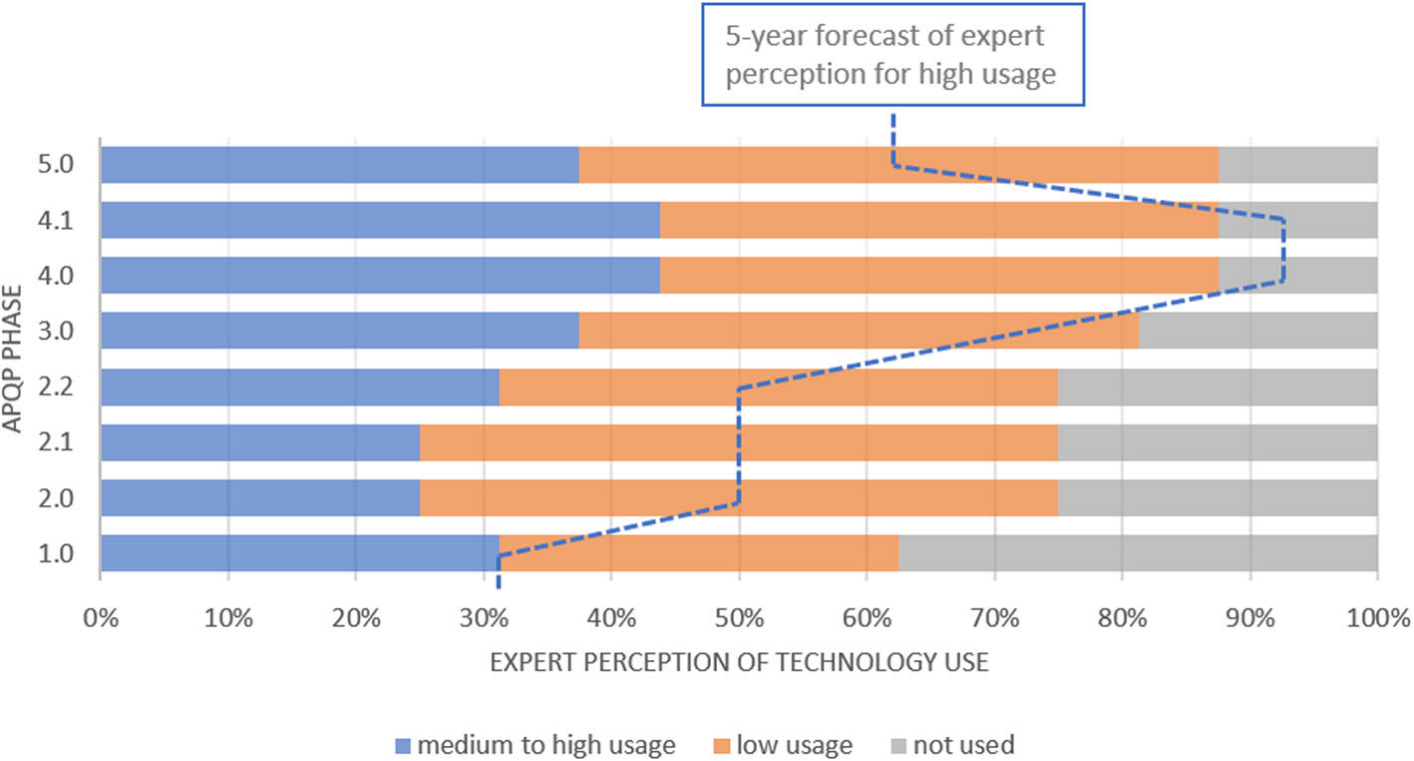
Adaptive Robotics There is a huge expectation, from the experts point of view, that adaptive robots without being widely used in phases 3 and 4 in the near future

It is almost unanimous that adaptive robots will be highly used in the next years in the phases 3 and 4, where product and process validation is carried out. In addition, adaptive robots use is also expected in corrective production actions to solve issues in phase 5 (Fig. 4).

**Fig. 5:**
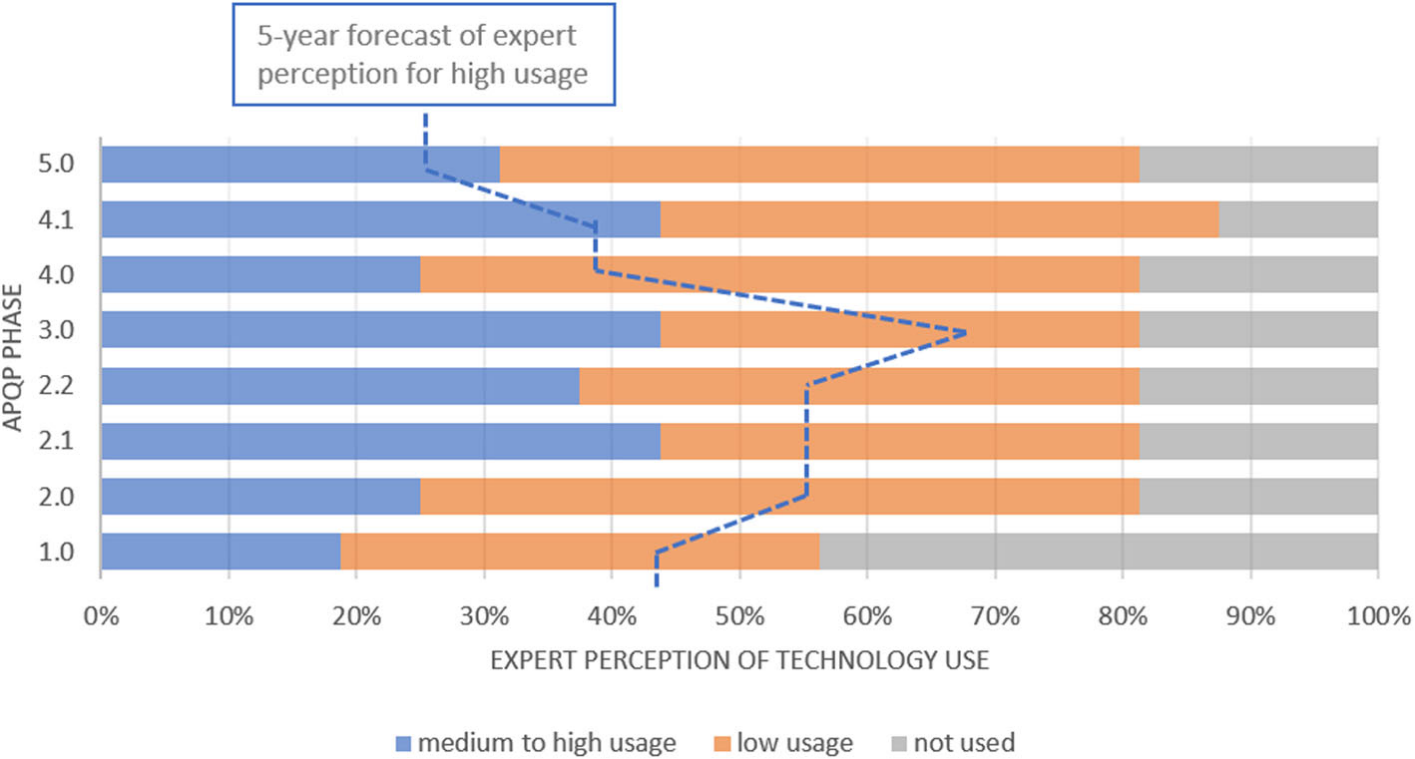
VR and AR These technologies should be used more in the phases of Product Development and Process Design in the next 5 years

VR and AR technologies are currently less used in phase 1 (Plan and Define Program) than the next phases of APQP. However, we expect a rise in theirs use over the next 5 years during this program definition phase. We emphasize that phase 3 can take advantage of these technologies, based on the usage ratio rise over the next 5 years (Fig. 5).

**Fig. 6:**
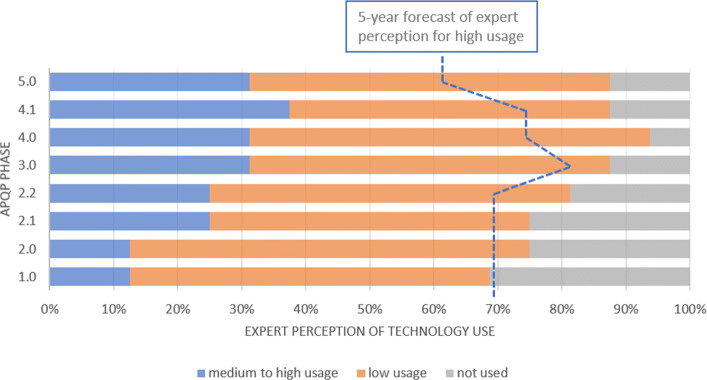
Cybersecurity This technology shows a huge rise of usage in all phases of the APQP

**Fig. 7:**
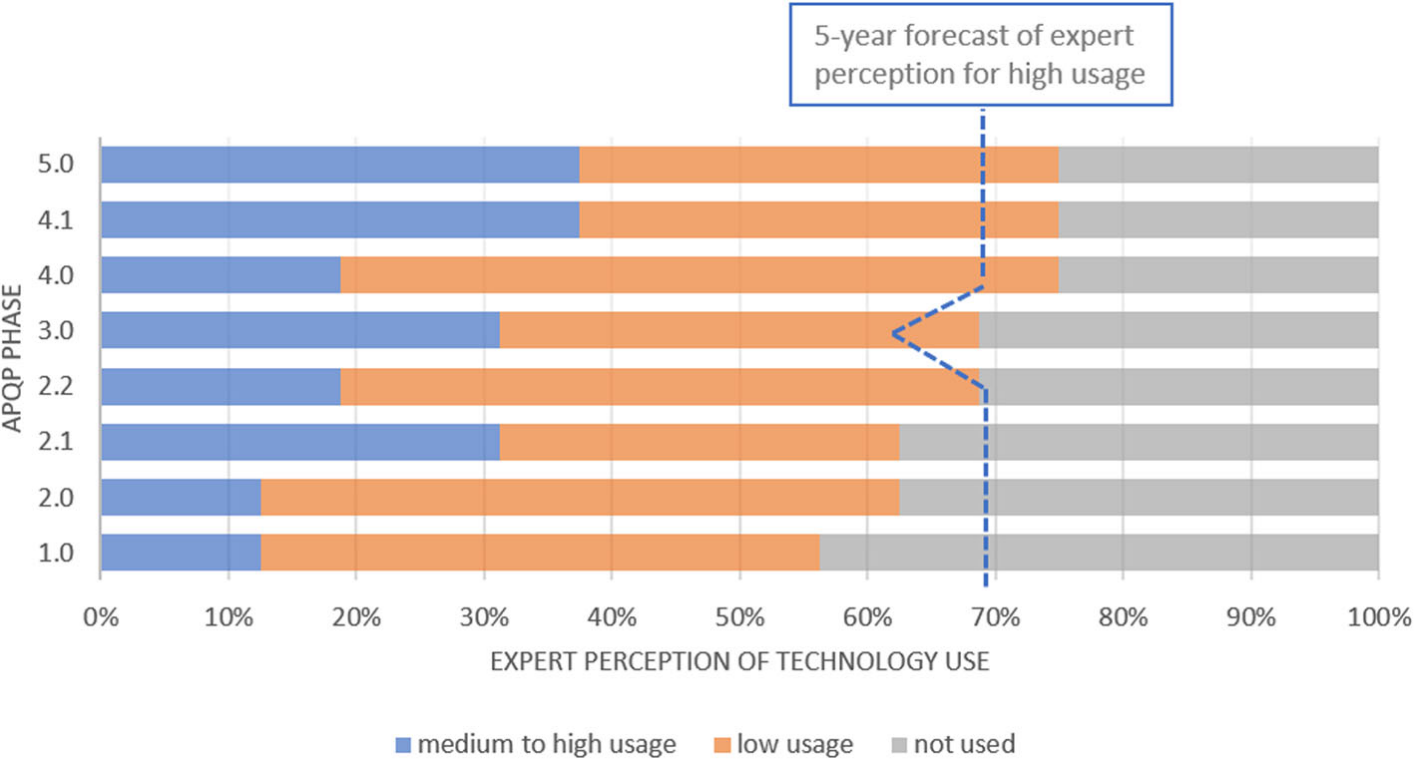
AI and Data Analytics This technology usage spike is similar to that of cybersecurity

Despite not being widely used today by the OEMs, both Cybersecurity and AI plus Data Analytics had a expectation of use in the next 5 years. Between 60 and 80 percent of experts said that these two technologies will be heavily used in all phases of APQP (Figs. 6 and 7). This may mean that the specialists understand the quantity of data and the complexity of the technology will increase and it will be useful in all the phases. By the way, we highlight the huge gap between the current and future situation for the use of these technologies presented in the survey. According to [32], traditional cybersecurity approaches may not work to protect Big Data. For this reason, the growth in the use of the two technologies together may be important to keep the Supply Chain widely connected with the OEM.

**Fig. 8:**
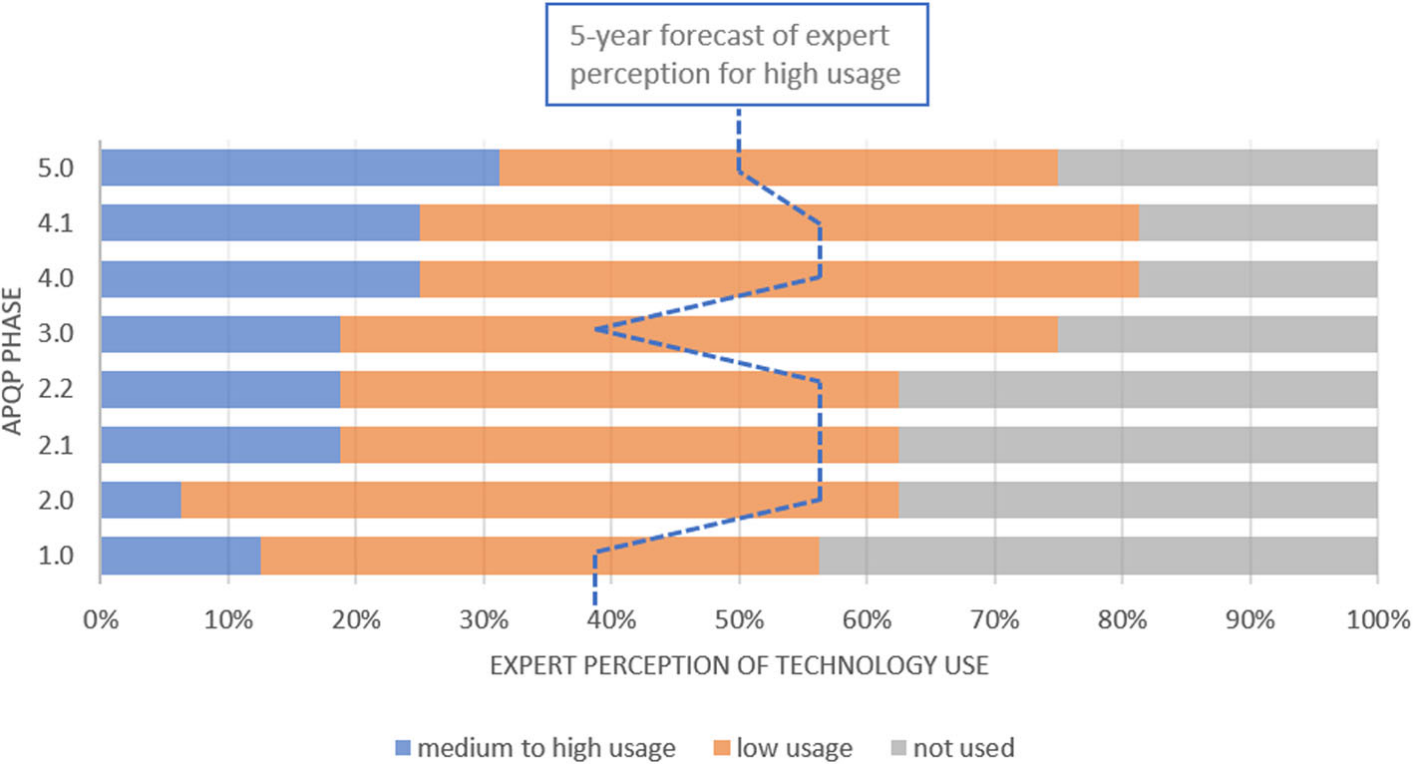
Cyber-physical systems This technology does not have an intense use today. It is possible to observe a rising in its use in phases 2 and 4

It is expected that OEMs will see an increase in the use of Cyber-physical infrastructure (Fig. 8). But Cyber-Physical Systems usage rise is not as sharp as that seen in AI and Cybersecurity answers. Cyber-physical systems are the merge between embedded software-intensive syshtems and global networks [33]. As cyber-physical systems evolve through the network of existing infrastructures with embedded information technology [33], this technology may be more perceived in the APQP with the adoption of others Industry 4.0 technologies.

**Fig. 9:**
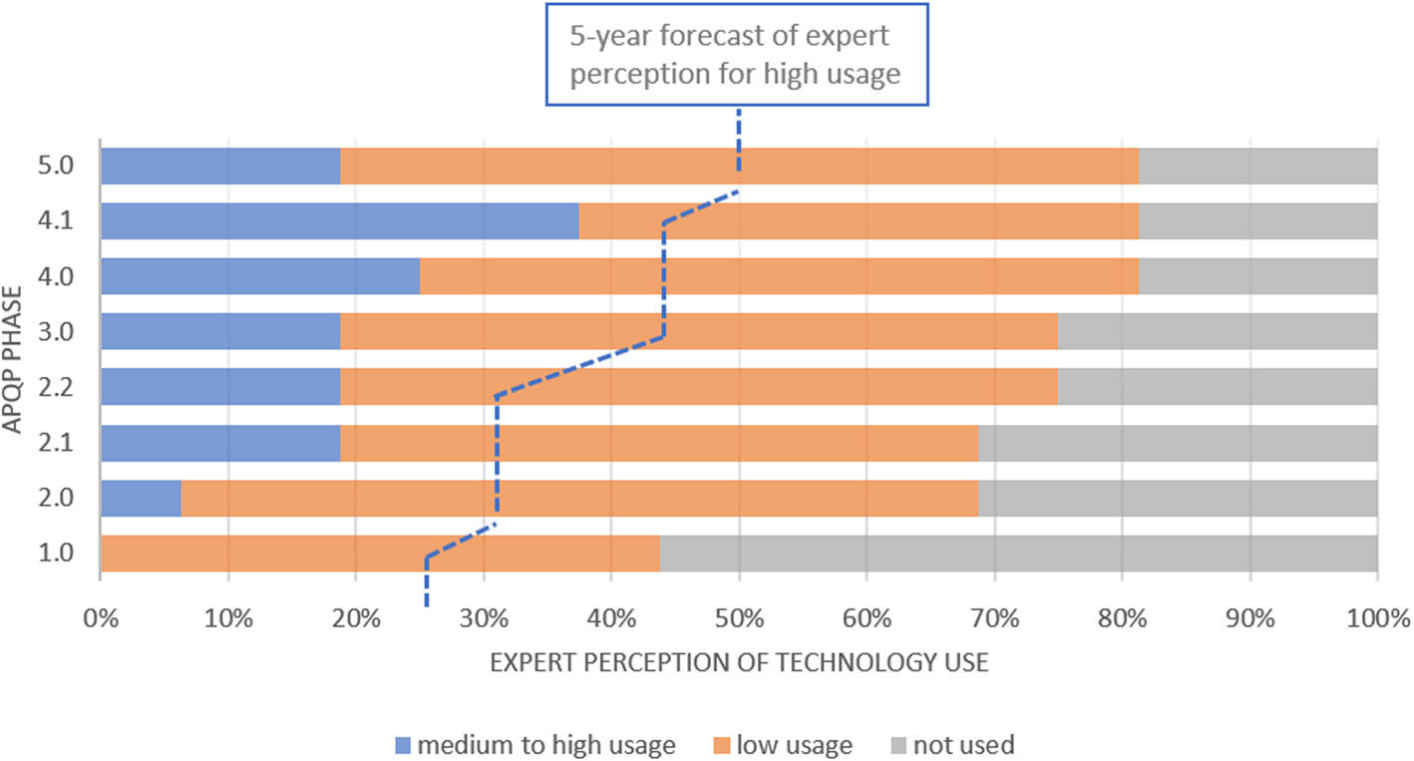
RTLS and RFID The technology does not appear to be widespread in the OEMs and its usage in the near future may be less than all the other technologies evaluated in this paper

The use of RTLS and RFID technology does not appear to be widespread in the OEM (Fig. 9. But when supply chain visibility becomes a reality, RFID is likely to be adopted across the supply chain [34]. One of the RFID value is rendering the supply chain visible and as a strategic capability within supply networks [35].

**Fig. 10:**
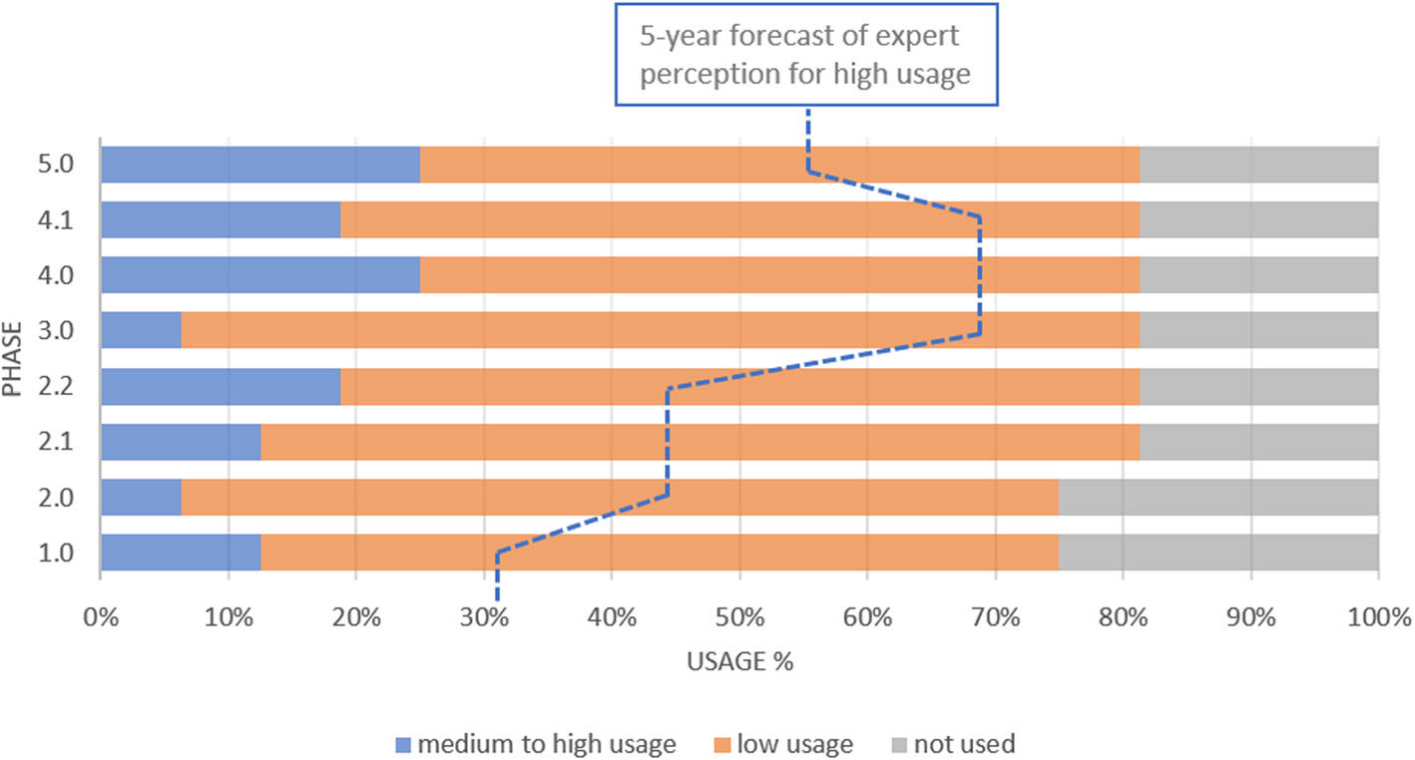
Additive Manufacturing This technology is little used today, but it was considered important for the scenario of 5 years ahead mainly in phases 3 and 4

Despite being considered little used today, additive manufacturing was considered important for the scenario of 5 years ahead, mainly for phases 3 and 4 when the engineering team design the process and validate the product and process, through PPAP (Fig. 10).

**Fig. 11:**
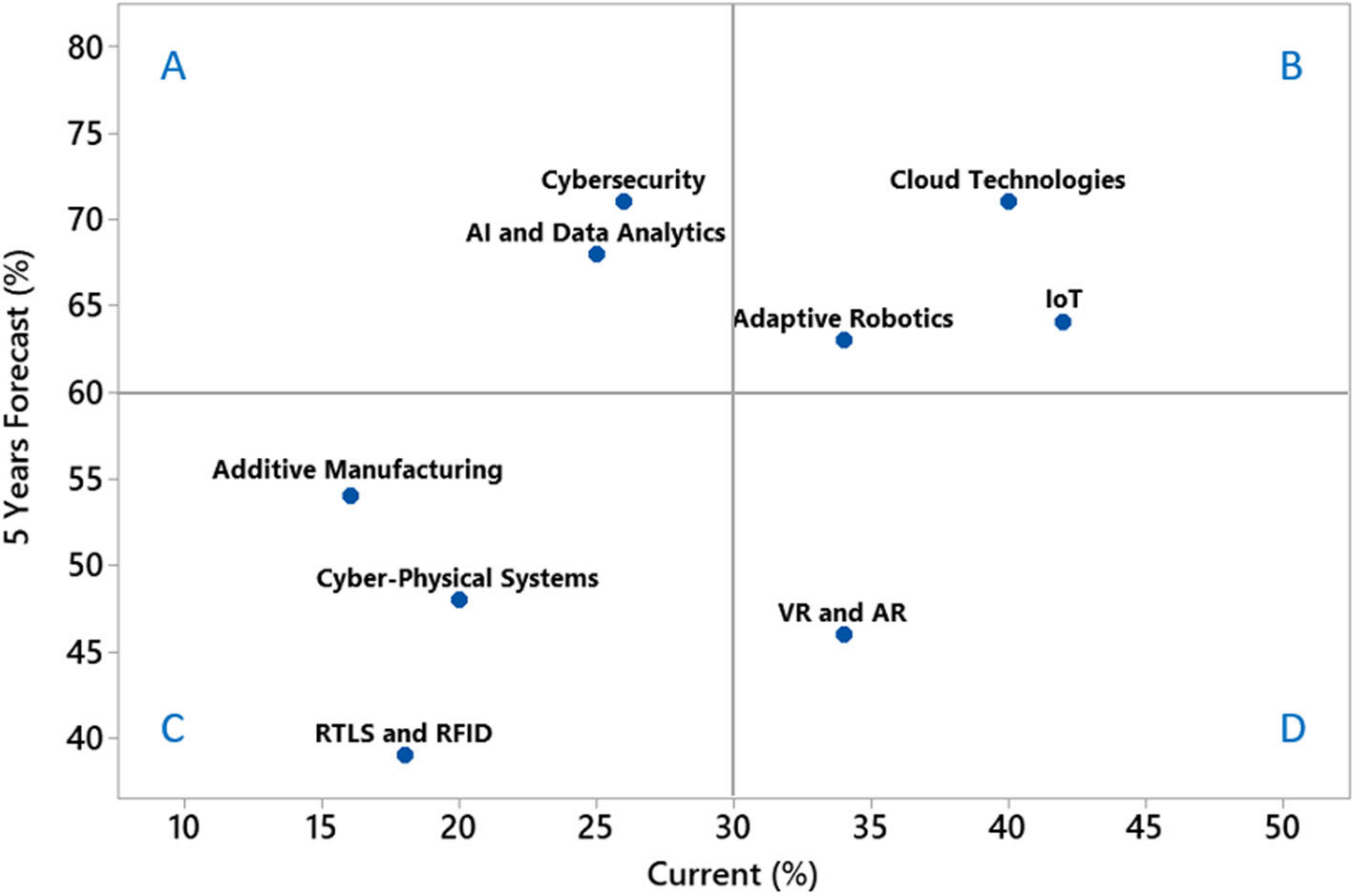
Scatter plot of the average current use versus average 5 years usage forecast of each Industry 4.0 Technology The limits of the quadrants (A, B, C and D) were defined based on the arithmetic mean of the responses related to the medium for high use of all technologies for all phases. They were used to group the technologies of Industry 4.0 in terms of the perception of their current use and the perspective of future adoption in the automotive OEM. The average values found were approximately 30% for the current use and 60% for the 5 years forecast

Figure 11 summarizes the results found for the current and future use of Industry 4.0 technologies by the Brazilian Northeast automotive OEM. First, Cybersecurity and AI with Data Analytics are the technologies with the potential of high adoption for the future scenario when compared to theirs current usage because they are located in the ‘A’ quadrant. Second, Cloud Technologies, IoT and Adaptive Robotics (quadrant ‘B’) are the most used technologies today and should present a rise in their use during the APQP in the 5 years. Despite being moderately used in practically all phases of APQP (Fig. 5), we do not foresee a rise in the use of VR and AR (quadrant ‘D’) as significant as the technologies found in quadrants ‘A’ and ‘B’. Lastly, Additive Manufacturing, Cyber-Physical Systems and RTLS plus RFID (quadrant ‘C’) were considered by specialists as the technologies less used today and that should not present a sharp rise of usage when compared with the technologies of the quadrant ‘A’. We highlight a sharp rise in the future adoption of additive manufacturing for the phases 3 and 4 of the APQP (Fig. 10) and for this reason it is closer to the quadrant ‘A’ limit when compared to the other technologies of quadrant ‘C’.

An association between the technologies that may mitigate the COVID-19 challenges presented in Table 1 and the technologies that should be most used for the next 5 years by Brazilian Northeast automotive OEM (Fig. 11, quadrants ‘A’ and ‘B’) may indicate the rise of resilience that the Brazilian Northeast supply chain would benefit from implementing the same technologies as the OEM. Lack of supply chain flexibility, lack of balance in supply and demand plus new consumer behaviors may be mitigated by the usage of AI and Data Analytics. Cloud technologies, IoT and Adaptive Robotics may be more present in solutions for communication issues, lack of security and poor infrastructure. Lastly, Cybersecurity may reduce the lack of access issue, considering the need for remote access to core systems.

## Conclusion

In this paper we presented a new method to evaluate the current situation and the future outlook for the usage of Industry 4.0 technologies by the automotive OEMs, identifying the issues that may be mitigated by the implementation of these technologies in the supply chain.

The developed survey was applied to evaluate the Brazilian Northeast automotive OEMs scenario from the perspective of manufacturing engineering experts on APQP. Firstly, we identified the technologies most used today, among them IoT, Cloud, Adaptive Robotics, VR and AR. Second, our results demonstrate the perspective of a significant spike in the use of Cybersecurity and AI plus Data Analytics throughout the APQP. Lastly, we relate how these five technologies may mitigate some of the challenges that emerged with the COVID-19 pandemic.

This study may provide insights for Tiers 1 and 2 to plan actions with the aim of implementing the main Industry 4.0 technologies to improve the SCR. The presented discussion is limited to the COVID-19 outbreak, which restricts the conclusion for other outbreak or supply chain disruptions conditions. Although the method can be applied to assess the scenario of the automotive industry in any region or country, the results presented in this paper are also limited to the Brazilian Northeast automotive industry and they can not be considered a snapshot of the national scenario.

Future studies may evaluate the challenges and the adoption of Industry 4.0 technologies in other locations, to understand which technologies may rise the SCR worldwide. It should also be used to evaluate the perspectives of the design and release engineering experts or compare different OEMs results.

## Data Availability

The datasets used and analysed during the current study are available from the corresponding author on reasonable request.

## Abbreviations

AI: Artificial Intelligence
ANFAVEA: Brazilian Association of Automotive Vehicle Manufacturers
APQP: Advanced Product Quality Planning and Control Plan
AR: Augmented Reality
OEM: Original Equipment Manufacturer
PDP: Product Development Process
RFID: Radio Frequency Identification
RTLS: Real-time Locating Systems
VR: Virtual Reality
